# Preventive Health Services Offered in a Sampling of US Emergency Departments, 2022–2023

**DOI:** 10.5811/westjem.18488

**Published:** 2024-07-17

**Authors:** Christopher L. Bennett, M. Kit Delgado, Melissa Pasao, Janice A. Espinola, Krislyn M. Boggs, Carlos A. Camargo

**Affiliations:** * Stanford University School of Medicine, Department of Emergency Medicine, Stanford, California; † University of Pennsylvania, Department of Emergency Medicine, Philadelphia, Pennsylvania; ‡ Massachusetts General Hospital, Department of Emergency Medicine, Boston, Massachusetts; § Harvard T.H. Chan School of Public Health, Department of Epidemiology, Boston, Massachusetts

## Abstract

**Introduction:**

In the United States, more chronic and preventive healthcare is being delivered in the emergency department (ED) setting. Understanding the availability of preventive health services in the ED setting is crucial. Our goal was to understand the availability of a subset of preventive health services in US EDs and explore how that has changed over time.

**Methods:**

In 2022–2023, using the National Emergency Department Inventory (NEDI)-USA, we surveyed a random 20% (1,064) sampling of all 5,613 US EDs. We asked directors of these EDs about the availability of and preference for 12 preventive health services, social worker availability, self-reported percentage of uninsured ED patients, and measures of ED crowding. We also asked about perceptions of barriers to implementing preventive health services in the ED. We used unadjusted and multivariable logistic regression models to compare service frequency in 2022–2023 to prior findings from 2008–2009 that represented a 5.7% random sampling of all EDs.

**Results:**

Among 302 responders to the 2022–2023 survey (5.4% random sampling, 28.4% response rate), 94% reported offering at least one preventive health service, with a median of five services. The most common service offered was intimate partner violence screening (83%), while the least common was routine HIV screening (19%). Seven services (eg, intimate partner violence, alcohol risk, and smoking cessation screening) had a higher odds of being offered in 2022–2023 than in 2008–2009; findings were unchanged in sensitivity analyses. A small proportion of directors opposed offering preventive health services. However, many expressed concerns that preventive health services in the ED would lead to longer lengths of stay (56%), increased costs to their ED (58%), a diversion of staff time from providing acute care (50%), or that their patients would not have access to adequate follow-up (49%).

**Conclusion:**

Nearly all EDs offer at least one preventive health service. Many offer multiple services; rates were higher than those identified in 2008–2009, in both unadjusted and multivariable models. Although limited by the response rate, this work provides the most recent and comprehensive snapshot of the type and frequency of a subset of preventive health services currently offered in US EDs.

Population Health Research Capsule What do we already know?

*A large proportion of all United States (US) healthcare is delivered in the emergency department (ED); this includes a growing amount of preventive care.*

What was the research question?

*To understand how the provision of a subset of preventive health services in US EDs has changed over time.*

What was the major finding of the study?

*Nearly all EDs studied (94%) reported offering at least one preventive health service, with a median of five services.*

How does this improve population health?

*This work provides the most recent snapshot of the type and frequency of a subset of preventive health services currently offered in US EDs.*

## INTRODUCTION

A large proportion of US healthcare is delivered in the emergency department (ED) setting.^1^ As an entry point into the healthcare system, EDs are providing an increasing proportion of both emergent and non-emergent (ie, chronic and preventive) care,^1^ ^,^ ^2^ in part due to insufficient access to primary care, population growth, and an aging population with increasingly complex medical needs. The recent end of the Public Health Emergency for COVID-19 and subsequent unwinding of the Medicaid continuous enrollment provision likely entails greater ED utilization for both chronic and preventive healthcare needs.^3^ However, it remains unclear what preventive health services are currently being offered in the ED setting and how this has changed over time. Findings from this study could help frame the changing landscape around ED reimbursements and incentive structures.

A study conducted in 2008–2009 engaged ED directors, determined the availability of a subset of preventive health services offered in a random sampling of US EDs, and characterized perceived barriers to implementing these services.^4^ Our objective was to provide an updated assessment of the availability of a subset of preventive health services following the onset of COVID-19 in EDs, given the expectation that resources are increasingly allocated to preventive care. The underlying goal was to offer insight into and contribute to the knowledge base supporting efforts to improve and optimize healthcare delivery within the ED setting.

## METHODS

We used the National Emergency Department Inventory (NEDI)-USA as a sampling framework for this study. The NEDI-USA is a comprehensive database of all non-federal, non-specialty US EDs; information available at the ED-level (eg, teaching hospital affiliation and annual visit volume) is updated annually.^5^ From NEDI-USA, we generated a random list of 1,064 EDs (≈20% of all US EDs).^5^ On the basis of this random list, directors were contacted up to three times (from Winter 2022 to Spring 2023) via e-mail or mail. Non-responders were contacted by trained research assistants via telephone.^4^

The instrument was a previously implemented survey (2008–2009) that characterized the availability of (and preference for) 11 preventive health services, ED-level social worker availability, self-reported percentage of ED patients who were uninsured, and measures of ED crowding in a 5.7% random sampling of US EDs (Appendix).^4^ If a service was not offered, the survey asked whether it could be offered given existing resources. Directors were also asked about perceptions of barriers to implementing preventive health services in the ED. In line with updated US Centers for Disease Control and Prevention (CDC) recommendations encouraging hepatitis C screening, the 2022–2023 survey also inquired about availability of routine hepatitis screening.^6^ Otherwise all other data elements, including the definition of crowding (ie, at least one of three CDC criteria: average waiting time of one hour or greater; left without being seen rate of 3% or more; or any time on ambulance diversion) were unchanged.^4^

In initial analyses, we summarized data with descriptive statistics (eg, counts, proportions, and medians with interquartile ranges [IQR]), and comparisons were conducted using statistical tests (eg, χ2 and Kruskal-Wallis tests). Logistic regression was then employed to assess the odds of preventive health services being offered more frequently in 2022–2023 than in 2008–2009. We also conducted sensitivity analyses with multivariable models adjusting for critical access hospital status. These data were summarized with odds ratios (OR) and 95% confidence intervals (CI). A two-tailed *P* < 0.05 was considered statistically significant. Analyses were completed in Stata 15.1 (Stata Corp, College Station, TX) and R Studio (https://www.R-project.org), and figures were created in R and Datawrapper release 0.4.6 (https://app.datawrapper.de/). This study was approved by the Stanford University Institutional Review Board and followed the Strengthening the Reporting of Observational studies in Epidemiology (STROBE) guidelines for observational studies.^7^

## RESULTS

Characteristics of responders, non-responders, and NEDI-USA overall are presented in the Table. The 302 responders (28.4% response rate) reflect a 5.4% random sampling of all 5,613 US EDs. With the exception of a higher proportion of responders representing small, rural, critical access hospital EDs (compared to NEDI-USA and non-responders), ED characteristics were otherwise similar. For context, characteristics of responders in 2008–2009 and 2022–2023 are presented in Supplemental Table 1; there were a similar number of total responders in 2008–2009 (277, 5.7% random sample of all EDs). In 2022–2023, responders were similarly more often from critical access hospitals than in 2008–2009; they also reported less ED social worker availability and—in the context of the previous passage of the Affordable Care Act—were less likely to report having more than 35% of their patients being uninsured.^8^

**Table.** Characteristics of responders, non-responders, and the National Emergency Department Inventory-USA.

	NEDI-USA, N = 5,613%	^*^Responders, n = 302% (95% CI)	Non-responders, n = 762% (95% CI)	*P*-value
Median annual visit volume (IQR)	20,000 (7,300–42,350)	14,216 (5,000–37,000)	16,572 (7,300–38,000)	0.07
Hospital type					
Teaching hospital	5	6 (4–9)	5 (3–6)	0.36
Trauma center				0.85
No	83	85 (80–88)	84 (81–86)		
Basic	8	7 (5–11)	7 (6–9)		
Advanced	9	8 (5–12)	9 (7–11)		
Critical access hospital	24	41 (36–47)	32 (29–36)	<0.01
Urban influence code				<0.01
Urban	66	51 (45–57)	57 (53–60)		
Large rural	14	13 (10–17)	17 (15–20)		
Small rural	20	36 (31–42)	26 (23–29)		
US region				0.06
Northeast	11	12 (9–16)	14 (11–16)		
Midwest	27	34 (29–40)	36 (32–39)		
South	43	34 (29–39)	37 (34–41)		
West	19	20 (16–25)	14 (11–16)		

* Confidence intervals not calculated for NEDI-USA because entire US population of EDs is included; values do not rely on an estimate. The 302 responders (28.4% response rate) reflect a 5.4% random sampling of all 5,613 EDs in the US; NEDI-USA reflects ED-level information from the 2019 NEDI-USA.

*CI*, confidence interval; *ED*, emergency department; IQR, interquartile range; *NEDI-USA*, National Emergency Department Inventory-USA.

**Figure 1.**
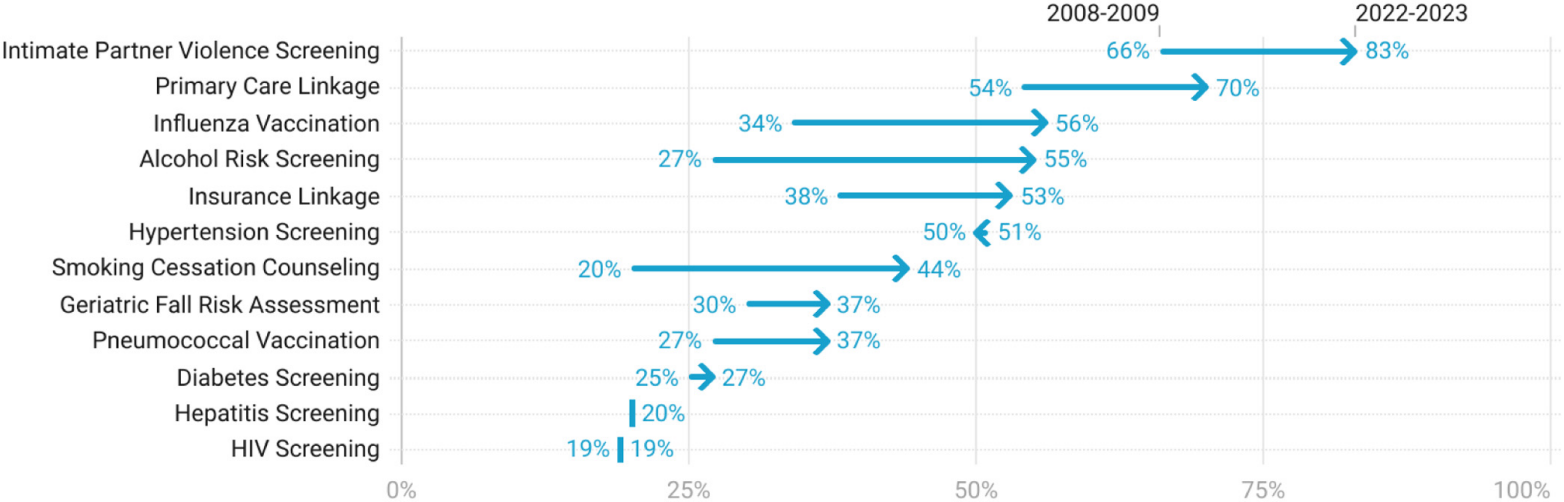
Availability of preventive health services, 2008–2009 and 2022–2023. The individual preventive health services are sorted by most frequent to least frequent. Hepatitis screening was not included in the 2008–2009 study; values reflect 2022–2023 data. Nearly all the preventive health services were more frequently offered in 2022–2023 than in 2008–2009.

Nearly all (94%) directors reported that their EDs routinely offer at least one of the 12 preventive health services, with a median (IQR) of 5 (3–7) services offered. The most common service offered was intimate partner violence screening, while the least common was routine HIV screening. Nearly all the preventive health services, except HIV and hypertension screening, were more frequently offered in 2022–2023 than in 2008–2009 (Figure 1). Seven of the services had higher odds of being offered in 2022–2023 than in 2008–2009; findings were unchanged after adjustment for critical access hospital status (Supplemental Table 2).

**Figure 2.**
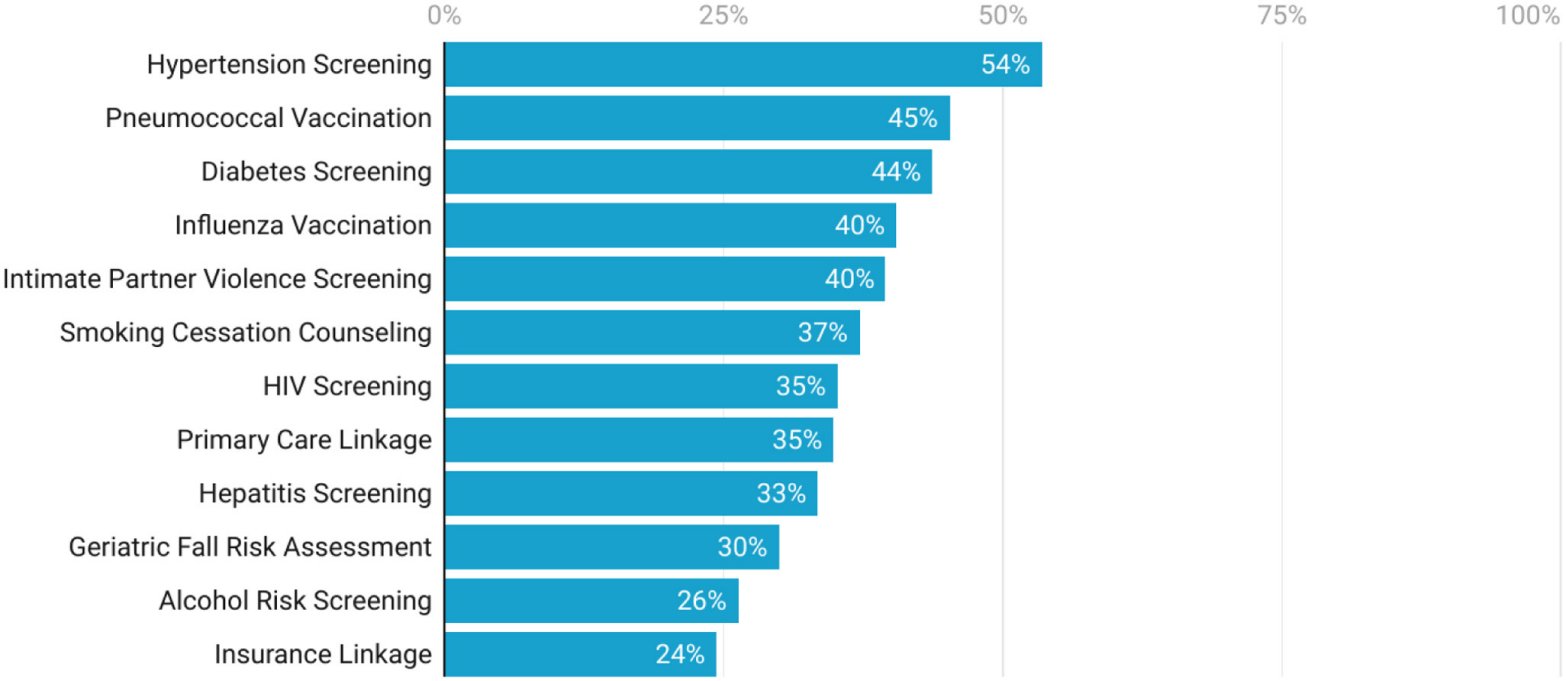
Ability to offer preventive health services with existing staff and funding in 2022–2023. Emergency department directors were asked whether they had a system in place to routinely provide a particular preventive health service. Those who reported “no” (ie, did not offer a particular service) were further asked whether they could already offer that service routinely given existing staff and funding. The individual preventive health services are sorted by most to least frequently reported as being possible to offer.

Further, among directors who reported that their ED did not offer a particular preventive health service, many still reported that resources were available to offer such services (Figure 2). When asked about their “first choice” of service they would most like to offer, alcohol risk screening, counseling, and referral was most common, while routine HIV and hepatitis screening were least common. Only a small proportion of ED directors thought that preventive health services should not be offered in the ED. However, as highlighted in the Supplemental Figure, many expressed concern (ie, strongly agreed or agreed) that offering preventive health services in the ED would lead to either longer lengths of stay (56%) or increased costs to their ED (58%), or would require a diversion of staff time from providing acute care (50%), or that their patients would not have access to adequate follow-up (49%).

## DISCUSSION

Among a random sampling of US EDs, nearly all offered at least one preventive health service, many currently have the resources to offer more, and only a minority of directors expressed the belief that preventive health services should not be offered in the ED setting. The results represent an increase in both the overall proportion of EDs offering at least one preventive health service and the median number of services offered per ED since 2008–2009.^4^ This finding is consistent with recent work demonstrating that EDs are providing a growing amount of chronic and preventive care in the US.^1^ ^,^ ^2^ A component of the results might be explained by the high proportion of responders from critical access hospitals and the unique mission these EDs have within their local communities. Reassuringly, adjustment for critical access hospitals did not materially alter the observed temporal difference.

Although we are unable to comment on the underlying reasons why (or why not) a particular ED offers a particular preventive health service, the reasons are likely multifactorial. Services that are mandated or strongly encouraged, compared to services that are neither, are likely more often offered. Further, services that are less time- and resource-intensive (eg, a series of screening questions compared to checking a hemoglobin A1c or performing a HIV antigen/antibody test) are also more likely to be offered. A component likely also depends on both the ED and its available resources, and the unique needs of the patient populations served in these EDs.

The observed changes occurred in the setting of the recent unwinding of Medicaid’s continuous enrollment provision, with the prospect that millions of Americans will lose—or have already lost—Medicaid coverage.^3^ This loss will likely translate into increased rates of ED utilization for both emergent and non-emergent (eg, chronic and preventive) care across the country. Given the staffing, crowding, and boarding crises in EDs, which were exacerbated by the COVID-19 pandemic, ED resources are expected to be further strained.^9^

## LIMITATIONS

Our work has several important limitations, among them our survey-based approach and response rate. A survey is the only feasible means to study this topic; these services are not typically billed for or trackable in any systematic national sample of US EDs. Reassuringly, our goal of obtaining a similarly sized random sampling of EDs as in 2008–2009 was met, and our response rate is consistent with recent work demonstrating survey-fatigue among healthcare workers during COVID-19 and lower survey response rates.^10^ For context, we provide detailed comparisons of how responders compare to non-responders, NEDI-USA, and to 2008–2009 responders. Given this limitation, and that both timepoints reflect distinct random samplings, we intentionally avoided formal pairwise comparisons between 2008–2009 and 2022–2023 responders. Instead, we incorporated a conservative approach using descriptive statistics and conducted regression to demonstrate that adjustment for critical access hospital status did not materially change our findings.

Second, we can only comment on availability for the preventive health services we considered; neither could we comment on the fidelity, comprehensiveness, or effectiveness of any of the preventive health services studied. Our objective was to provide an updated assessment of the availability of a subset of preventive health services in the ED setting, with the goal of highlighting the increasing amount of non-emergent care being provided. Third, given our goal of using the same survey vehicle to facilitate comparisons against prior work we cannot comment on the extent of, or ED-level differences in, these services. These issues are important and merit future investigation but are beyond the scope of the current work. These issues, and work focused on cost effectiveness, reimbursements, and financial incentives of preventive health services offered in ED settings, are the focus of current and future efforts by our group. Despite these limitations, our findings represent the most current and comprehensive snapshot of the availability and frequency of preventive health services currently offered in US EDs.

## CONCLUSION

Nearly all. EDs in the United States offer at least one preventive health service. Many EDs offer multiple services, and rates were higher than those identified in 2008–2009 in both unadjusted and multivariable models. Although limited by the low response rate, this work provides the most recent snapshot of the type and frequency of a subset of preventive health services currently offered in US EDs.

## Supplementary Information





