# Supplementary material for: Preventive Health Services Offered in a Sampling of US Emergency Departments, 2022–2023
Source: West J Emerg Med. 2024 Jul 17;25(5):823–7. doi: 10.5811/westjem.18488 (PMC11418861; doi:10.5811/westjem.18488)
Supplement: Supplementary file 1 [file wjem-25-823-s001.docx]

National Survey of Preventive Health Services in U.S. Emergency Departments

Thank you for completing this year’s National ED Inventory (NEDI)-USA 2021 Survey!


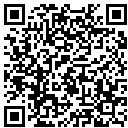


Online Survey

We are conducting a short survey to evaluate ED directors’ attitudes towards offering preventive health services in the ED. Your ED was selected from a random sample of the nations’ EDs through the National ED Inventory (NEDI) database compiled by the Emergency Medicine Network (EMNet – www.emnet-usa.org). While there have been many publications regarding these types of services in the academic setting, it is unknown what services are currently being offered by most EDs and what barriers ED directors perceive towards offering these services. You will be asked to answer a series of questions regarding preventive health services available in your ED as well as barriers surrounding the accessibility of these services to ED patients.

Your participation will take approximately **3 to 4 minutes** to complete. Please send your answers to us in the attached, **pre- paid envelope**. We also welcome responses by *[blinded]* or scan the QR code **above**.

We kindly request that this survey be completed **within 1 week** of receipt.

If you prefer to opt-out of further contact regarding this study, please confirm by email or online at the REDCap link.

If you have any questions, concerns or complaints about this research, its procedures, risks and benefits, contact [*blinded]*

We are deeply grateful to you and for your time in completing this brief survey and participating in this research.


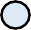

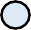

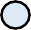

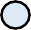

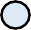

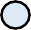

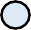

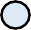

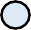

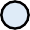

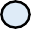

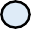

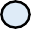

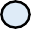

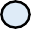

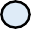

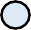

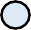

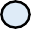

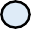

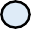

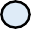

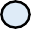


**Please confirm your hospital information below:**

1. What is the name of your hospital/ED?
2. What is the mailing address of your hospital/ED?
3. If known, what is your hospital/ED NEDI ID?

**Please select the most appropriate answer to each question.**

35% or greater

25-34%

15-24%

5-14%

<5%

8) What percentage of your ED patients are uninsured (self-pay)?

20% or greater

10-19%

1-4% 5-9%

0%

7) What % of the year is your ED on ambulance diversion status?

5% or greater

3-4%

1-2%

<1%

6) What percentage of registered ED patients leave without being seen by a clinician?

2 hours or greater

1 hour

30-59 minutes

<30 minutes

5) What is the average time from triage sign-in to being placed in an ED treatment bed for patients arriving through the waiting room?

24 hours

16-23 hours

8-15 hours

1-7 hours

0

4) How many hours per day are social work services available in your ED?

***** Survey continues on other side

Thank you! Please contact us at *blinded* with any questions.

# Please circle either “yes” or “no” in response to the questions regarding each ED preventive service.

| ED Preventive Health Service | Is there a system in place that routinely  performs this service in your ED? | If not, could you offer this service routinely  with existing staff and funding? |
| --- | --- | --- |
| A) Alcohol risk screening, counseling, and referral for all patients with  drinking-related complaints | Yes / No | Yes / No |
| B) Diabetes screening and referral for nondiabetics with ED glucose > 160 | Yes / No | Yes / No |
| C) Geriatric fall risk PT/OT assessment and intervention | Yes / No | Yes / No |
| D) HIV screening and referral (non-needle stick injury related) | Yes / No | Yes / No |
| E) Hepatitis screening and referral (non-needle stick injury related) | Yes / No | Yes / No |
| F) Hypertension screening and routine referral of patients with BP >  160/90 | Yes / No | Yes / No |
| G) Influenza vaccination screening, and, if needed, provision of the  vaccine | Yes / No | Yes / No |
| H) Intimate partner violence screening and, if needed, safety assessment  and referral for all women | Yes / No | Yes / No |
| I) Pneumococcal vaccination screening, and, if needed, provision of the  vaccine. | Yes / No | Yes / No |
| J) Reliable linkage of patients without a usual source of care to primary  care | Yes / No | Yes / No |
| K) Screening for insurance and linkage of eligible uninsured patients to  insurance programs | Yes / No | Yes / No |
| L) Smoking cessation counseling and referral for all smokers | Yes / No | Yes / No |

| **Of all the services above unavailable in your ED, which services**  **would you most like to offer given your patient population?** | **1st Choice:** | A | B C D | E F G H I | J K | L |
| --- | --- | --- | --- | --- | --- | --- |
| Please rank up to three choices by circling the letter corresponding to **2nd Choice:** | | A | B C D | E F G H I | J K | L |
| the service above.  Circle “None” if you do not want to offer any. | **3rd Choice:**  **None** | A | B C D | E F G H I | J K | L |

**Please respond to each of the following statements below by circling a response (1-5) from the scale below:**

(1= Strongly Disagree) (2= Disagree) (3= Neutral) (4= Agree) (5= Strongly Agree)

| a) “I worry that implementing preventive services would increase ED patient length of stay.” | **1** | **2** | **3** | **4** | **5** |
| --- | --- | --- | --- | --- | --- |
| b) “I worry that implementing preventive services would lead to increased financial costs to my ED due to lack of reimbursement for added tests, vaccines, and/or counseling.” | **1** | **2** | **3** | **4** | **5** |
| c) “Our patients would not have adequate access to follow-up to make some of these ED preventive services effective.” | **1** | **2** | **3** | **4** | **5** |
| d) “I do not think that preventive services should be offered in the ED.” | **1** | **2** | **3** | **4** | **5** |
| e) “I worry that implementing preventive services in the ED would divert the time of physicians and nurses away from providing acute care, possibly leading to worse patient outcomes.” | **1** | **2** | **3** | **4** | **5** |


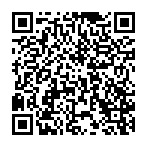


Future Compensated Research Interest Form

If yes, please indicate your interest and provide your contact information at: *[blinded]* **or** Scan the QR Code:

**Are you interested in participating in future compensated research?**

# We welcome your comments and suggestions! Please feel free to write in the space below, or to contact us at *[blinded]*
