# Supplementary material for: Preventive Health Services Offered in a Sampling of US Emergency Departments, 2022–2023
Source: West J Emerg Med. 2024 Jul 17;25(5):823–7. doi: 10.5811/westjem.18488 (PMC11418861; doi:10.5811/westjem.18488)
Supplement: Supplementary file 2 [file wjem-25-823-s002.docx]

**Supplemental Table 1:** Characteristics of Responders in 2008-2009 and 2022-2023

|  | **Responders** | | | |
| --- | --- | --- | --- | --- |
|  | **2022-2023 (n=302)** | | **2008-2009 (n=277)** | |
|  | **%** | **95% CI** | **%** | **95% CI** |
| **Median annual visit volume, (IQR)** | 14,216 (5,000-37,000) | | 21,024 (7,884-37,668) | |
| **Hospital type** |  |  |  |  |
| Teaching hospital | 6 | (4-9) | 8 | (4-11) |
| Critical access hospital | 41 | (36-47) | 26 | (21-32) |
| **Urban Influence Code** |  |  |  |  |
| Urban | 51 | (45-57) | 57 | (51-63) |
| Urban adjacent | 25 | (21-31) | 24 | (19-29) |
| Large rural | 6 | (4-9) | 7 | (4-10) |
| Small rural | 18 | (14-22) | 12 | (8-16) |
| **U.S. Region** |  |  |  |  |
| Northeast | 12 | (9-16) | 13 | (9-17) |
| Midwest | 34 | (29-40) | 41 | (35-47) |
| South | 34 | (29-39) | 28 | (23-34) |
| West | 20 | (16-25) | 18 | (13-23) |
| **Uninsured patients >35%** | 10 | (7-14) | 34 | (29-39) |
| **Crowded** | 43 | (37-48) | 46 | (40-52) |
| **ED social worker available** | 64 | (59-70) | 76 | (71-81) |

***Legend****: Responders in 2022-2023 reflect a 5.4% random sampling; responders in 2008-2009 reflect a 5.7% random sampling; corresponding NEDI-USA information from the 2019 (for 2023-2023) and 2007 (for 2008-2009) NEDI-USAs.*

***Abbreviations****: CI (confidence interval), ED (emergency department), IQR (interquartile range), U.S. (United States)*

**Supplemental Table 2.** Logistic Regression Results for Comparison Between 2008-2009 and 2022-2023, Unadjusted and Adjusted for Critical Access Hospital Status

|  | **Unadjusted** | | | | **Adjusted for Critical Access Hospital Status** | | | | |
| --- | --- | --- | --- | --- | --- | --- | --- | --- | --- |
| **Preventive Health Services** | **OR** | **95% CI** | | **P-value** | **OR** | **95% CI** | | **P-value** | |
| Intimate Partner Violence Screening | 2.49 | 1.67 | 3.69 | <0.001 | 2.70 | 1.80 | 4.04 | | <0.001 |
| Primary Care Linkage | 1.94 | 1.38 | 2.74 | <0.001 | 1.97 | 1.39 | 2.79 | | <0.001 |
| Influenza Vaccination | 2.50 | 1.78 | 3.51 | <0.001 | 2.37 | 1.68 | 3.35 | | <0.001 |
| Alcohol Risk Screening | 3.27 | 2.30 | 4.65 | <0.001 | 3.54 | 2.47 | 5.09 | | <0.001 |
| Insurance Linkage | 1.86 | 1.33 | 2.60 | <0.001 | 2.03 | 1.44 | 2.86 | | <0.001 |
| Hypertension Screening | 0.96 | 0.69 | 1.34 | 0.83 | 0.90 | 0.65 | 1.26 | | 0.56 |
| Smoking Cessation Counseling | 3.14 | 2.16 | 4.56 | <0.001 | 3.11 | 2.13 | 4.53 | | <0.001 |
| Geriatric Fall Risk Assessment | 1.33 | 0.94 | 1.89 | 0.11 | 1.42 | 0.99 | 2.03 | | 0.06 |
| Pneumococcal Vaccination | 1.58 | 1.11 | 2.26 | 0.01 | 1.47 | 1.02 | 2.12 | | 0.04 |
| Diabetes Screening | 1.13 | 0.78 | 1.64 | 0.53 | 1.06 | 0.73 | 1.56 | | 0.75 |
| HIV Screening | 0.97 | 0.64 | 1.47 | 0.87 | 1.01 | 0.66 | 1.53 | | 0.98 |

***Legend****: For 2008-2009 outcomes, we incorporated critical access hospital status from the 2007 NEDI-USA. For 2022-2023 outcomes, we incorporated critical access hospital status from 2019 NEDI-USA. For both models, 2008-2009 was used as the reference.****Abbreviations****: CI (confidence interval), OR (odds ratio)*

**Supplemental Figure**: Director-Reported Barriers to Implementing Preventive Health Services in the ED, 2022-2023

**
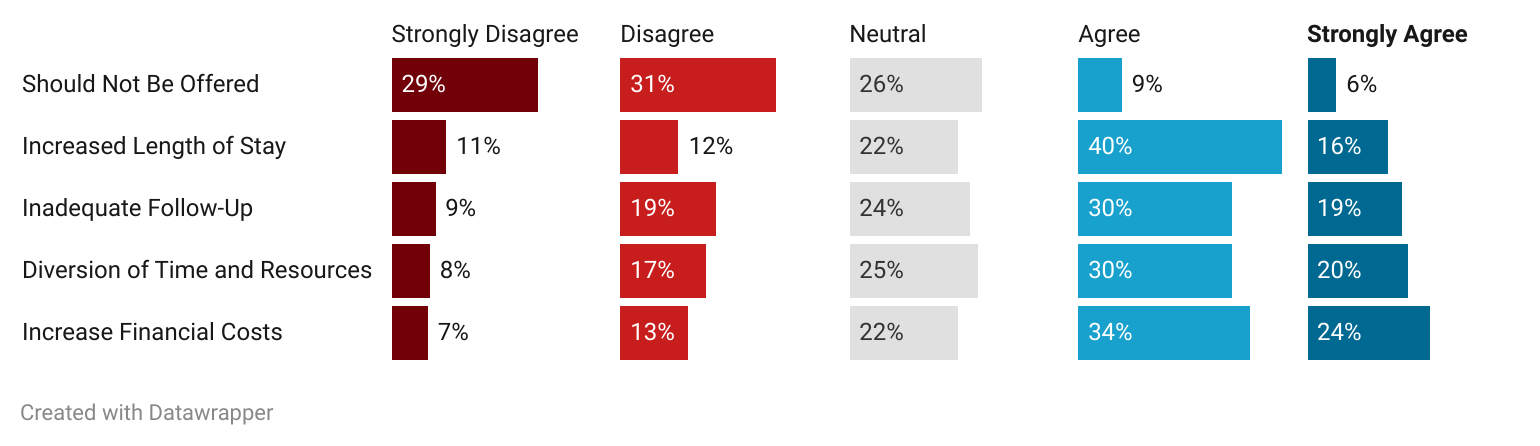
**

***Legend****: ED directors were presented with five questions and a corresponding Likert scale (1-5; strongly disagree-strongly agree) regarding their thoughts on offering preventive health services in the ED and perceived barriers to implementation. As described in the* ***Appendix****, “should not be offered” corresponds to “I do not think that preventive health services should be offered in the ED.” “Increased length of stay” corresponds to “I worry that implementing preventive services would increase ED patient length of stay.” “Inadequate follow-up” corresponds to “Our patients would not have adequate access to follow-up to make some of these preventive services effective.” “Diversion of time and resources” corresponds to “I worry that implementing preventive services in the ED would divert the time of physicians and nurses away from providing acute care, possibly leading to worse patient outcomes.” “Increased financial costs” corresponds to “I worry that implementing preventive services would lead to increased financial costs to my ED due to lack of reimbursement for added tests, vaccines, and/or counseling.”*

***Abbreviation****: ED (emergency department)*
